# Paediatric on-call consultants’ learning within and beyond the objectives of a coherent CPD program

**DOI:** 10.1186/s12909-022-03895-6

**Published:** 2022-12-14

**Authors:** Daniel Holmgren, Maria Skyvell Nilsson, Per Wekell

**Affiliations:** 1 Department of Paediatrics, Skaraborg Hospital, Skövde, Sweden; 2 Institute of Clinical Sciences, University of Gothenburg, Gothenburg, Sweden; 3 Department of Health Sciences, University West, Trollhättan, NU-Hospital Group, Trollhättan, Sweden; 4 Department of Paediatrics, NU-Hospital Group, Uddevalla, Sweden

**Keywords:** CPD, Learning, Coherent program, Paediatrician, On-call consultant

## Abstract

**Background:**

Evaluations, using questionnaires, of a two-year long CPD program for on-call consultant paediatricians, showed that the overall objective of the program was largely met. We stipulate that the coherency of the CPD program contributed to the learning. To gains a deeper understanding of the participants learning within and beyond the overall objectives of the program, we decided to conduct an interview study enrolling participants from the first two CPD courses.

**Methods:**

Nine experienced paediatric consultants were interviewed 1-4 years after completing a coherent two-year long CPD program, focusing on what and how they learned. The interviews were audio-recorded and transcribed as text, analysed, and categorised using qualitative content analysis.

**Results:**

What the participants learned: improved medical competences, greater confidence in the role of an on-call consultant, better understanding of the role of an on-call consultant and importance of professional networks. Several categories were outside the overall objective, at personal level: an understanding of one’s own and other’s competences, taking responsibility for one’s own CPD and managing things one does not know. At professional level: more secure as an individual and with colleagues.

How it was learned: relevant objectives, preparatory material and case discussions were important. Participants learned by preparing, repeating, reflecting, and participating actively, and by applying what they learned in clinical practice. The participants learned from one other over a period of two years, when they also got to know one another and created networks. A safe learning environment imposed demands and enabled participants to define their competence and learn accordingly.

**Conclusions:**

This study describes what and how on-call consultant paediatricians learned during a coherent two-year long CPD program. The learning took place within and beyond the framework of the overall objectives. The study suggests that evaluation methods based on objectives may be blind to important areas of learning and need to be combined with qualitative methods that examine a broad impact of learning. Taken together, the analysis of what and how the participants learned shows that they were better equipped to work as consultant on call and deal with the things they did not know.

**Supplementary Information:**

The online version contains supplementary material available at 10.1186/s12909-022-03895-6.

## Background

As a result of rapid medical developments and increasingly specialized and advanced healthcare, the need for continuing professional development (CPD) of Doctors have become increasingly clear during the past few decades [1, 2]. It has also become more important for fully qualified Doctors to collaborate and work in teams. For this reason, knowledge of learning, communication, organisation, and leadership needs to be strengthened [3].

The CPD of Doctors is the natural continuation of basic medical education and specialist (postgraduate) training, but it differs in several respects. Both the basic medical education and specialist training of Doctors are strictly regulated. This is not the case for CPD in many countries, where CPD primarily has been designed with individual Doctors’ continuous learning in mind.

From mainly focusing on continuous medical education (CME), learning after specialisation currently aims at continuing professional development (CPD). CME is dealing with knowledge and skills of medical practice, whereas CPD reflects the wider context in which the fully qualified doctor works, and includes an assessment of the individual’s competence, professional development, collaboration, and the creation of a good learning environment [1, 4].

In 2003, to enhance the quality of CPD, the World Federation for Medical Education (WFME) formulated the first global standard. It has been revised and approved by the federation’s general assembly several times, most recently in April 2021 (WFME website: http://www.wfme.org). In 2006, the standard was adopted by the EU as a policy for the CPD of Doctors and was jointly sanctioned in 2011 by the Swedish Medical Society and the Swedish Medical Association [5].

The way in which the CPD of Doctors is organised, implemented, and evaluated differs substantially in different parts of the world. In Sweden, CPD is not compulsory, but the Swedish Medical Association is driving the CPD question and has proposed that CPD should be regulated by law and implemented in the form of individual CPD plans that are followed up and reviewed at regular intervals [6].

As the demand for and on CPD activities has increased, interest in the pedagogic knowledge of adult learning has risen. Research has highlighted different theories to understand the best way of learning as an experienced Doctor. In summary, adults learn best when the learning is relevant to their clinical practice, focus on problem solving, values experience, and is based on active participation, exchange between colleagues and provides opportunities to transfer the knowledge into practice [4].

To improve the effectiveness and strengthen adult learning principles of the CPD activities for consultant paediatricians on-call in western Sweden, we created a coherent CPD program for those that needed to update their competence or were about to take on the task [7].

The rational for developing a coherent curriculum are particularly strong for paediatric CPD as the speciality is broad and highly specialised at the same time, comprising most adult specialities, and in addition includes areas specific for paediatrics, for example neonatology and adolescence medicine.

The aim of the program was that participants after the program should be able to work independently as on-call paediatric consultants during a weekend at an emergency hospital with inpatient care for children and adolescents. The methods we used to develop the CPD program are grounded in established pedagogic knowledge and the principles of adult learning [8, 9]. The curriculum formulation started with a survey of educational needs among the target group, which laid the foundations of the overall objectives and content of the program, and the objectives of the individual learning modules. The learning strategy of the program was based on learning in four stages: 1) preparation by reading the literature; 2) training on solving clinical problems in case discussions and simulation sessions; 3) an evaluation of the learning and 4) return to clinical work [7, 8].

We were also aware that adults learn in different ways and by reflecting on previous experiences when they encounter new situations [10, 11]. This highlighted additional factors that were important in the design of the program, such as:

- The survey of the learning needs of the target group identified several areas that lay outside pure medical knowledge and skills
- The program and learning objectives were introduced and anchored among paediatricians and paediatric subspecialists in the region as well as among heads of paediatric departments and representatives of the academia.
- The extensive and complex overall objectives of the program necessitated a coherent program over a longer period.
- The participants’ experiences, competences and skills were regarded as learning resources as they, both formally and informally, were both teachers and students.

The CPD program was coherent in several ways, it was contextually defined with shared overall objectives and learning methods and ran over a period of two years with the same participants who functioned as both educators and learners. A summary of the program is presented in the Methods section. Course I has previously been reported in detail [7].

The first course [7], was run in 2010-2012 with 16 participants, half of whom also took part as teachers in their areas of paediatric expertise, while the second was run in 2013-2014, with 22 participants, three of whom also participated as teachers.

Evaluations, using questionnaires, during and in the end of the first two courses showed that the overall objective of the program was largely met. We stipulate that the coherency of the CPD program contributed to the learning. To gains a deeper understanding of the participants learning, we decided to conduct an interview study enrolling participants from the first two CPD courses for on-call consultant paediatricians.

## Aim

The aim of the present study was to investigate how a coherent CPD program for on-call consultant paediatricians contributed to the participants’ learning, within and beyond the over-all objectives of the program.

### Specific objectives

To investigate,

- What did they learn?
- How did they learn?

## Methods

The study was conducted through semi-structured interviews of a purposive sample of paediatricians that completed the CPD program for on-call consultants. The interviews were analysed using qualitative content analysis with a combined inductive and deductive approach [12–14].

### Participants

To give the participants time to reflect on and experience how the CPD program impacted their clinical practices, participants from two courses were recruited, 1-4 years after the completion of the individual course. The CPD program comprised 16 days of face-to-face learning, with the same time spent on preparation, during a period of two years. A summary of the overall objectives, content, and criteria for successful completion of the CPD program is given in Table 1. The Course II had the same structure and learning modules as Course I besides three additional learning modules: one day on Infectious Diseases/Immune Deficiencies and a half-day Workshop on Online Medical Applications.

**Table 1:** Overall objectives of the CPD program [7], content of Course I [7] and a summary of the examination criteria that were applied to Courses I and II

**Overall objectives of the CPD program** [7]			
Upon completion of the two-year course, you should be able to: • Assess the competence of the doctor on-call and adapt your own work to match this • Guide and support the doctor on-call to work in a satisfactory, independent way and assume primary responsibility as needed • Collaborate with and set appropriate boundaries for other levels of care and specialists/specialties • Collaborate with social and other authorities, as well as the police • Manage the primary phase of disaster and unexpected events, as well as contact with the press • Develop your own basic paediatric knowledge and clinical skills relating to conditions and situations that may occur during a weekend on call • Take the initiative and responsibility for updating your basic knowledge and skills relating to conditions and situations that may occur during a weekend on call			
**Content of Course I** [7]			
First year	Subject	Face to face (days)	Preparation (days)
Term 1	Introduction	½	
	APLS (Advanced Paediatric Life Support)	2	2
	Nephrology/Cardiology	1	1
	Diabetes/Immunology	1	1
Term 2	Neonatal simulation	1	1
	Paediatric intensive care	1	1
	Gastroenterology/Metabolic disorders	1	1
	Ethical issues	1	1
	Written home-examination		1
Second year			
Term 3	Transplantation/Commission formulation	1	1
	Child abuse and maltreatment	1	1
	Oncology	1	1
	Neurology	1	1
Term 4	Neonatology	1	1
	Child in catastrophe/Media/Search of information	2	1
	Written home-examination, Lecture		1
	Completion of program	½	
Total		16	16
**Criteria for successful completion of the Courses I and II**			
Compulsory learning modules; successful completion of the APLS course; neonatal simulation training; teaching assignment; home examinations I and II; participation in the consultant on-call rotation (including certification of competence from head of department)			

To get a broad description of the learning, we included participants of both genders, with different long experience from each level of care in the hospital organization. Furthermore, the interviews were conducted after varying lengths of time regarding when the participant completed the course. The inclusion of participants was based on a purposive sampling from the two courses, according to gender, experience as an on-call consultant (long-short), type of hospital (secondary-tertiary level) and length of time since course completion (long-short).

Eleven on-call consultant paediatricians were selected and asked to consent, five men and five women, agreed to participate and were interviewed. One declined due to lack of time. One of the interviewed on-call consultants worked abroad and was therefore excluded.

The number of interviewed participants was considered sufficient to answer the research question and to transfer knowledge to other contexts with regard to the methodology [15]. Background data on the nine included on-call consultants are shown in Table 2.

**Table 2:** Background data on the nine interviewed paediatric on-call consultants included in the study

**Order of interviews**	**Gender** Male (M) Female (F)	**Physician since** (years)	**Qualified paediatrician since** (years)	**Consultant on call since** (years)	**Time of interview after completing the course** (years)	**Course**	**Hospital level** Secondary (S) Tertiary (T)
1.	M	31	23	10	0.8	II	T
2	F	20	10	1,5	0.9	II	T
3	M	27	24	13	3.2	I	T
4	M	23	17	15	0.9	II	S
5	F	16	9	4	3.3	I	S
6	M	26	25	10	3.3	I	S
7	F	16	9	9	3.7	I	S
8	F	23	16	7	1.6	II	S
9	F	18	16	9	1.5	II	S
**Median**							
Course I		21	16.5	9.5	3.3		
Course II		23	16	9	0.9		

### Interviews

The interviews were conducted individually, between December 2015 and November 2016. All the interviews were conducted by one of the authors (MSN) and undertaken at a time and in a place that was convenient to the participants and free from distraction. The interviewer was not involved in the development or implementation of the program. Seven interviews were carried out at physical meetings and two on a digital platform. The interviews lasted between 30 and 50 minutes. Before the interviews, the participants were informed about the study, including the fact that participation was voluntary, that they could withdraw at any time and that they could decline to answer any question.

An interview guide was used (Supplemental Table A) and the interviewer explained that the interview focused on what they learned and how they learned, during and after the course.

The development of the interview guide was based on the answers to the open questions as part of the course evaluations at the end of the course I and II, respectively. The interview guide was designed with open-ended questions to give the participants the opportunity to freely describe their experiences of the program and its contribution to their knowledge. The questions were formulated in a way that they would enable rich descriptions of the participants experiences in relation to the overall objectives. No specific validation of the questions was made. The interviews were digitally recorded, using a voice recorder, and transcribed verbatim, producing 117 printed pages of text.

#### Analysis

The complete text was read several times by the authors (DH, MSN) to make sense of the data. To capture manifest and latent content of the text, the degree of abstraction in the interpretation of the data was increased stepwise, during the analysis. The text was reduced into meaning units, with the aims and objectives of the study and the interview guide in mind [12]. The meaning units were read several times by all the authors, after which they were abstracted to condensed meaning units in a first step and condensed meaning units with an interpretation of the underlying meaning in a second step. They were then abstracted into codes. The codes were sorted under “*What was learned”* and *“How it was learned”* respectively. The analysis procedure is illustrated by an example in Supplement Table B. The codes that responded to the question “*What was learned?”* were further analysed, categorised, and the categories and subcategories were sorted under the overall objectives of the CPD program (deductive approach). During this process, it became clear that several categories and subcategories fell outside the overall objectives of the CPD program. To try to capture all the aspects of “*What was learned?”,* including those that fell outside the overall objectives of the CPD program, all the codes were reanalysed, creating new categories and subcategories which were sorted under the twelve impact categories of CPD suggested by Allen LM et al. [16] (deductive approach).

To catch the learning within and beyond the over-all learning strategies of the program the codes that responded to the question *How it was learned* formed the basis for creating categories and subcategories using an inductive, text-driven approach, to search for patterns [12, 13]. We chose to end the analysis of the data on a descriptive level, using categories and subcategories in the result section corresponding to a high interpretation degree and low abstraction level, according to Graneheim et al. 2017 [14].

## Results

The transcribed text was reduced into a total of 202 meaning units, 432 condensed meaning units, and 360 condensed meaning units with an interpretation of the underlying meaning. Finally, the condensed meaning units with the same underlying meaning were merged and abstracted into 309 codes.

### What was learned!

A total of 160 codes responded to the question *What was learned* and were further interpreted and abstracted to categories which were sorted under the overall objectives of the CPD program, illustrated in Table 3.

**Table 3:** Responses to the question of what was learned, categorised, and sorted under the overall objectives of the course

**Categories covered by the overall objectives of the program**
**Assess the competencies of the Doctor on-call and adapt your own work to match this**
• Strengthened own clinical competence
• Ways of working as an on-call consultant
• Support the Doctor on-call
**Guide and support the Doctor on call to work in a satisfactory, independent way and assume primary responsibility as needed**
• Support the Doctor on-call
• Strengthened consultant on-call competences
• Define own competence
**Collaborate with and set appropriate boundaries with other levels of care and specialists/specialties**
• Consensus within the region
• Consensus within the region when managing paediatric emergency situations
• Importance of networks
• Understand own and other’s competence
**Collaborate with social and other authorities, as well as the police**
• See the child’s perspective in relation to its parents
• Take care of child maltreatment
**Manage the primary phase of disaster and unexpected events, as well as contact with the press**
• Manage media contacts
• Apply the disaster contingency plan
• Manage emergency situations
**Develop your own basic paediatric knowledge and clinical skills relating to conditions and situations that may occur during a weekend on call**
• Extended competence
• More in-depth competence
• Methods for seeking knowledge
• Manage medical emergency situations
• Take responsibility for own continuing professional development (CPD)
**Take the initiative and responsibility for updating your basic knowledge and skills relating to conditions and situations that may occur during a weekend on call**
• Take responsibility for my own CPD
• Update my knowledge
• Method for quickly searching for knowledge
• Manage medical emergency situations
**Categories that fell outside the overall objectives of the program**
**Knowledge, skills and experiences**
• Ways of managing things I don’t know
• Security in the role of on-call consultant
• Confidence
• Less stress in and when presented with emergency situations
• The importance of personal contacts and networks
• Apply modern pedagogics
• Apply knowledge that improves colleagues and my practice
• Understand the role of an on-call consultant at different levels of care
• Confirms that I can work as an on-call consultant
• Understand other professional’s competences
• The importance of one’s own competence and CPD
• Define my competence
• Sometimes the objectives do not match my own expectations
**Miscellaneous**
• Time for new ideas, share thoughts and reflect
• Confidence shown by my manager gave me the chance to control preparations
• The course is unable to deal with my anxiety relating to administrative problems such as gaps in the on-call schedule and the lack of beds for sick children in the region
• Reference cases
• *“When I learn something, I also want to learn it in detail, but that wasn’t really the target”*
• You need to keep learning the whole time

This categorisation revealed that the participants learned according to the overall objectives of the program, such as: *extended competence, ways of working as an on-call consultant* and the *importance of networks* (Table 3). Other categories of what they learned within the framework of the overall objectives, were: *understanding one’s own and other’s competences, supporting the doctor on-call, taking responsibility for one’s own CPD* and the importance of a *consensus within the region when it comes to the management of paediatric emergency situations*.

It emerged that 19 of the 49 categories fell outside the overall objectives of the CPD program.

Many of the categories that fell outside the programs overall objectives related to areas such as self-awareness and professional relationships. Categories such as: *defining my competence* and *ways of managing the things I don’t know*, as well as the *importance of networks,* support the hypothesis that the participants found strategies for managing the role of on-call consultant and that the things they learned, during the course and in the application of this knowledge in their clinical work, *confirm that I am capable of working as an on-call consultant*. The participants are aware of what they know, what they do not know and how to find out about the things they do not know.

The additional analysis, interpretation, and categorisation of codes of what was learned, which was performed to try to capture all the aspects of what was learned, including those categories that fell outside the overall objectives of the CPD program in the firs analysis, resulted in 28 categories and 35 subcategories. These categories and subcategories were sorted under the twelve impact categories of CPD, suggested by Allen LM et al. [16], illustrated in Table 4.

**Table 4:** Responses to the question of what was learned, categorised, and sorted under the twelve impact categories of continuing professional development (CPD), suggested by Allen LM et al. [16]. The second column indicates whether the impact category corresponded to the overall objectives of the program

	**Learning objective**
**Knowledge**	
**Medical knowledge**	**Yes**
Updated	
More in-depth	
Broader	
**Knowledge of the duties of an on-call consultant**	**Yes**
Duties of an on-call consultant	
The competence that are needed to work as an on-call consultant	
**Practice change**	
**Use material from the course in clinical work**	**Yes**
Literature	
Treatment guidelines	
**Change in clinical work**	**Yes**
Return to case discussions from the course	
How to communicate in complicated situations	
Adapt the level of investigation to the fact that you are on call	
**Skill, ability, competence and performance**	
**Strengthen on-call competences**	**Yes**
Generally	
Manage emergency situations	
Perform practical procedures	
Manage the media	
Search for knowledge	
Manage disasters and crises	
Communicate with Doctor on-call	
**Other competences**	
Define one’s own competence	
Apply pedagogic principles of adult learning	
Manage difficult clinical situations	
**Confidence**	
**More secure**	
In the function of on-call consultant	
Managing things I don’t know	
**Self-confidence**	
In managing difficult clinical situations	
**Trust in my own competence**	
**Attitudes**	
**Approaching working as an on-call consultant**	**Yes**
Top-class competence is needed	
I can’t know everything – the importance of networking for patient safety	
Leading and supporting the primary on-call Doctor	
**Take responsibility for one’s own CPD**	**Yes**
**The importance of sharing ideas and reflecting with others**	
**Being able to see the child’s perspective even in conflict with its parents**	**Yes**
**Career development**	
**Starting to work as an on-call consultant**	**Yes**
**Continuing to work as an on-call consultant**	**Yes**
**Deeper understanding of the role of on-call consultant**	**Yes**
**Networking, collaboration and relationships**	
**Collaborating with and supporting the primary on-call Doctor**	**Yes**
**The importance of networks**	
**The importance of knowing one another**	
**Understanding colleagues’ different competences**	
**Consensus in managing clinical problems**	
**Understanding the role of the on-call consultant at different levels of care**	**Yes**
**User outcomes**	
**A way of working so that**	
Things are better for children	Yes
The Doctor on-call does a better job	Yes
Others do a better job	
**Intention to change**	
Not relevant	
**Organizational change**	
**Identify and deal with shortcomings at your own department**	
Create new memos	
Greater patient security in emergency situations	
**Course pedagogics**	
Applied in the training of consultants at your own department	
Contribute to a learning environment which facilitates new recruitment and gets colleagues to stay at the clinic	
**Personal change**	
**More secure**	
As a person	
Together with colleagues	
**A feeling of being able to learn new things**	
**Deeper relationship with colleagues**	
**Scholarly accomplishments**	
Not relevant	

The additional analysis and categorisation of data revealed that it was possible to sort all the categories and subcategories under one of Allen’s categories. The categorisation according to Allen emphasized*: the importance of knowing one another, trust in my own competence,* and *confidence* in *managing things I don’t know*.

Other aspects represented by categories that fell outside the overall objectives in the first analysis that could still be categorised according to Allen were: *apply pedagogic principles of adult learning,* course pedagogic applied in *the training of consultants at own department* and working in a way so that: *the Doctor on-call does a better job, others do a better job* and *things are better for children.* This indicates that experienced paediatric on-call consultants take responsibility for their colleagues and the department both during and outside on-call time. The fact that the course participants experienced support from their department was illustrated in the statement: “*the support of my boss made it possible to take control over the preparations”.*

### How it was learned!

A total of 149 codes responded to the question *How it was learned*. These codes were interpreted and abstracted into 15 categories and 44 subcategories. The further analysis, using an inductive approach, revealed that most of the answers could be related to specific stages during the CPD program. We therefore chose to sort the categories and subcategories under the different stages of the program: during the preparations prior to the face-to-face learning, during the face-to-face learning, in the clinical work during and after the course and generally, with no direct link to any specific stage during or after the course, Table 5.

**Table 5:** Responses to the question of how it was learned, categorised, and sorted under the different stages of the CPD program

**During the preparations**
**Preparations**
Read and repeat
Have time – take time
Have access to relevant literature
Be motivated
Take responsibility
Reflect
**Would have liked**
More time for preparation
Less literature
Guidance on what was most important to read
**During the face-to-face learning**
**Case discussions**
Listen to colleagues
Discuss with others
Learn from one another
Feedback
Confirmation that I am thinking along the right lines
Be motivated
**Would have liked …**
Subspecialist expert in small groups, in some cases
Summary of knowledge status, in some cases
** Creating networks**
**Continuity – getting to know one another**
Dare to show the things you don’t know
Informal conversations
Openness and security
Demands were imposed
**Teachers’ and participants’ competences**
High level of competence
Long experience
Different competence and experience
Role models
**Simulation training**
Media training
Crisis role play
Advanced Paediatric Life Support (APLS)
Search for knowledge
**Participating as a teacher**
**Reflecting over tasks, competences, and learning**
Thinking in new ways
Building on own knowledge and experience
Formulate the assignment
Try to understand more than the course objective
**Motivation**
Relevance
Continuity
Participation
**Examination**
Group-based home exam
Evaluation of scenarios of APLS
**Would have liked …**
Relevant questions on APLS exam
**In the clinical work during and after the course**
**Clinical work during learning and learning during the clinical work**
Start to work as an on-call consultant
Return to course literature, cases, and notes
Repeat
Continue to build on experience from the course
Create teaching opportunities at your own clinic
Contact colleagues when there is something I don’t know
**Would have liked …**
Follow-up and repetition of the course
Network for on-call consultants
**Generally**
**Reflect on your own competence in the role of an on-call consultant**
Competence in relation to the Doctor on-call
Competence in relation till colleagues
**Course pedagogics**
Clearly defined purposes and objectives
Case discussions, interactive pedagogics
Continuity
**Start from the child’s perspective**
**Support from employer**
**Would have liked …**
The course leaders to have the same picture as I had of what I was going to learn, in some cases

#### During the preparations for face-to-face learning

The answers relating to the way the participants learned underlined categories such as: *taking one’s own responsibility* and being *motivated.* Categories such as *having access to relevant literature*, *having time – taking time, reading, and repeating* and *reflecting* also emerged. This indicates that the participants understood that they needed to continuously learn and take their own responsibility for their learning, but also that they were motivated to learn. Having time to read relevant literature gave them the opportunity to reflect on and integrate new information in relation to previous experience, which resulted in the emergence of new knowledge [11].

#### During the face-to-face learning

In the interviews, all the participants gave answers that could be interpreted and sorted under the categories, that they had learned through *simulation sessions* and *case discussions*. A quotation from one of the participants in the interview study, referring to the APLS course, demonstrates that learning through simulation sessions is relevant and effective: *“You can’t wait to get going on a difficult problem and being able to learn so much in such a short time is fantastic!”.* The participants’ appreciation of case discussions is illustrated by subcategories such as: *Learn from one another*, *Discuss with others* and *Confirmation that I am thinking along the right lines*.

*Examination* in the form of group-based home exams was a kind of learning in itself and this is illustrated by a quotation from a participant: “*There were some tests we had to take and so the three of us sat and discussed things and this naturally impacted the learning*”.

The participants understanding and respect for one another’s perspective was illustrated by the collaboration, both during the course and when they contacted one another in the clinical work. The important effect this has on learning is illustrated by the categories they learned through, *teachers’ and participants’ competences, continuity – getting to know one another* and *creating networks*.

This CPD program was two year long, which gave the participants the opportunity to *reflect on assignments, competences and learning* over a relatively long period. It also made it possible to reflect on their new experience and what they had learned. Or, as one participant put it, *“If you have been working for a number of years, it’s easier to think along the same old lines. It is therefore beneficial to devote time to thinking about areas you don’t often consider in slightly new ways”.*

#### In the clinical work during and after the course

All the interviewees gave answers that could be interpreted and sorted under the category *clinical work during learning and learning during clinical work.* This indicates that the participants, both during and after the course, continued to learn by applying their new-found knowledge, competence, and experience in their clinical work, such as *starting to work as a consultant on-call* and *repeating, returning to literature, cases, and notes* during the clinical work.

#### General

This CPD program contained no formal pedagogic education, but all the participants experienced the program pedagogics over a period of two years and 11 of the total of 38 participants applied the pedagogics as teachers. The pedagogics had an impact on learning, and this is underlined by the fact that more than half the participants gave answers that could be interpreted and sorted under *course pedagogics*. The category *participating as a teacher* also indicates that the participants learned by applying the learning methods in the course and being evaluated by their colleagues on the course which, from a pedagogic learning perspective, corresponds to the highest level of “does” according to Miller’s “Framework for clinical assessment” [17, 18].

Another category that emerged as important for learning was *reflecting on one’s own competences in the role of an on-call consultant,* both in relation to the on-call Doctor and in relation to other colleagues. Several participants also gave answers that could be sorted under the category *support from my employer* which indicates that this played an important part in their learning.

The category *starting from the child’s perspective* illustrates a central theme for the entire course, integrated in all the learning procedure, but it was given special attention during the learning modules dealing with ethics and child maltreatment.

## Discussion

The curriculum of the present CPD program for on-call consultants was inspired by Kern´s curriculum formulation, (instrumental learning theories) and Kolb’s experiential learning model [8, 9]. Kolb’s learning model is a learning strategy, that is relevant in medical education, since it focuses on learning of competencies and practising of skills in concrete and specific situations [8, 19]. However, the extensive and complex overall objectives of the program required a broader type of learning to develop and keep the program continuously relevant for a complex and rapidly changing clinical scope. Thus, learning strategies such as Schon’s reflective learning theories [11] and learning for an unknown future according to Bowden and Marton [10] alerted us to be open for a variation of learning in the program. The result in the present study indicates that the participants learned beyond the overall objectives and applied learning strategies outside the intention of the program.

### What the participants learned

A central point in both the AMEE’s guide to effective CPD and the WFME’s global standard for quality improvement within CPD is to evaluate the learning in relation to the objectives of the program [1, 4]. The results of what the participants reported that they learned, indicates that the learning strategy was effective and confirmed that the overall objectives of the program was largely met. In addition, the participant developed knowledges and attitudes beyond the overall objectives of the program, which relate to areas such as self-awareness and professional relationships. These aspects could be categorised and sorted under the impact categories suggested by Allen et. al. [16]. This is interesting since a deductive approach often create problems with getting all the categories to fit into the explanatory model [14]. Nevertheless, the results of the additional data categorisation highlighted the importance of including evaluations of CPD programs with qualitative evaluation methods that also capture the effects that fall outside the objectives of a program formulated in advance [16].

#### Coherency of the program – contributed to what the participants learned

We were not able to explicitly determine the significance of the coherency of the program on participants learning. However, we claim that categories such as, *getting to know one another, importance of networks, ways of managing the things I don’t know*, that emerged in the analyses of the data, was a consequence of this aspect of the program design.

The fact that the participants were given both time and support from their employer, to prepare for the learning modules, and attend the complete program, made the participants motivated, and gave them the opportunity to take personal responsibility for their own CPD. This learning can be described by *Motivational models* [19].

The two-year program imposed demands on the participants to prepare and participate actively in the case discussions, but it also made it possible for the participants to get to know one another and learn from each other, and dare to expose what they didn’t know, in a safe setting. They were aware of what they know, what they do not know and how to find out about the things they do not know. Such learning is related to*, Humanistic theories* and *social theories of learning* [19].

### How the participants learned

The inductive approach to the analysis of the participants’ responses to how they learned revealed categories representing a variation of learning strategies and theories.

The reading assignment and preparation to the face-to-face learning was mandatory, but we had not any specific check-ups on how the participant completed the assignments. Based on our observations during the face-to-face sessions such as the content of the discussions as well as notes and underlining’s in the course material etc., we maintain that most of the participants prepared thoroughly. Moreover, we believe that the whole program’s pedagogy contributed to the participants’ motivation to read the material with a deep learning approach, according to Bowden and Marton [10].

It was clear that the participants appreciated the simulation sessions. Previous studies have demonstrated that simulation is an effective pedagogic method for clinical learning [20]. The participants’ appreciation of case discussions is in line with what others have found [21]. However, it has not been possible, to empirically demonstrate that learning by case discussions is more effective than other methods [21]. On the other hand, the case discussions in this CPD program were based on real-clinical cases in which the participants faced realistic situations and problems which they had to resolve together, under the guidance of experts in the respective fields. This can be labelled a kind of “real case, non-practical simulation”. It gave them experience from authentic situations and the opportunity to apply new knowledge to which they could relate when they returned to clinical work a learning related to Kolb’s learning cycle [8]. On the other hand, the face-to-face learning where the participants had to solve clinical problems in collaboration with each other by “reflection-in-and-on-action”, may be seen as related to *Reflective* and *Transformative learning theories* [11, 19].

#### Coherency of the program – contributed to how the participants learned

Several of the participants in the CPD programs were among the most experienced and well-qualified sub-specialists in Sweden, while others were clinically experienced but less specialized. As a result, they developed a mutual respect for one another’s knowledge, competences, and skills.

Thus, the collective knowledge, competence and experience among the participants were extensive before the program started, knowledges and experiences that were articulated and further developed into new knowledge by the participants in collaboration with the educators during the program and in their clinical work. Such a learning can be linked to *Social Constructivist theories* of learning [19]. That the program was coherent over two years gave the participants the opportunity to apply what they learned during the program in their clinical practice and at the same time gain new knowledge in their clinical practice that they could use in the case discussions during the program. This learning strategy may be seen as linked to Kolb’s experiential learning cycle [8].

In order to meet the difficult and complex demands that a pediatric on-call consultants needs to address, we strived to integrate theory and practice, pedagogy and research in the CPD program. Such an approach may be seen as linked to a learning through variation, when facing a complex and unknown future or “to prepare for the unknown by means of the known”, according to Bowden & Marton [10, 22].

## Methodological considerations

Even if the study is based on a purposive sampling, the number of interviewed participants was limited and, for this reason, the results cannot be said with certainty to be representative.

The answers in the interviews gave a rich description of the participants experiences, within and beyond the overall objectives [15] and matched the results of the regular evaluations of the CPD program by open questions, which indicates that the interviewees still represented the target group in general.

## Summary

### What was learned

The categorization of interview data in accordance with both the overall objectives for the program and Allen’s categories, supports the view that the participants were better equipped to work as on-call consultant paediatricians which was the overall aim of the program. The additional analysis and categorisation according to Allen revealed that the participants obtained an understanding of their own and others’ ability, were able to take responsibility for their own CPD and to use their knowledge, competences, and skills to improve both their co-workers and their practice. At the same time, it also revealed that the participants had become more confident in their role as on-call consultants, both as individuals and in collaboration with their colleagues, and that they had developed an effective strategy for managing the things they did not know.

### How it was learned

Relevant objectives, preparatory material and case discussions were extremely important. Through preparations, repetition, reflection, and the application of newly acquired learning, the participants learned from active participation in the program and in their clinical work. The coherent program gave the participants the opportunity to learn from one another and take advantage of one another’s experience over time, as well as getting to know one another and creating networks. This contributed to a safe learning environment, in which they were not afraid to show the things they didn´t know which also imposed demands.

## Conclusion

The present study shows that the curriculum and learning methods in this coherent CPD program created an opportunity for learning in accordance with and outside the framework of the overall objectives for the program. The qualitative evaluation of the CPD program revealed effects that have an important impact on job satisfaction, professional development, and a good working environment [16]. The results indicate that evaluation methods that are based solely on objectives of a program may be blind to important areas of learning and that methods of this kind need to be combined with qualitative methods that examine learning in a broad sense. Taken as a whole, the analysis of what and how the participants learned during and after the program shows that they were better prepared to deal with the things they did not know and for an unknown future [10].

## Supplementary Information

**Additional file 1: Supplemental Table A.** The interview guide used during the interviews of the nine paediatric on-call consultants.

**Additional file 2: Supplement Table B.** An example of the analysis procedure for the interpretation of the transcribed interview text, using qualitative content analysis.

## Data Availability

The datasets used and/or analysed during the current study available from the corresponding author on reasonable request.
