# Supplementary material for: Paediatric on-call consultants’ learning within and beyond the objectives of a coherent CPD program
Source: BMC Med Educ. 2022 Dec 14;22:865. doi: 10.1186/s12909-022-03895-6 (PMC9749295; doi:10.1186/s12909-022-03895-6)
Supplement: Supplementary file 1 — Additional file 1: Supplemental Table A. The interview guide used during the interviews of the nine paediatric on-call consultants. [file 12909_2022_3895_MOESM1_ESM.docx]

Supplemental **Table A**. The interview guide used during the interviews of the nine paediatric on-call consultants.

**Interview guide**

- What did you think about the course?
- What was good/bad respectively?
- In what way did this experience contribute to your competence as an on-call consultant?
- Can you describe a situation in which this competence was useful, working as an on-call consultant?
- What does this competence mean to you personally?
- What does this competence mean to the role as an on-call consultant?
- What do you think this competence means to the on-call consultant?
- What does this competence mean to the patient?
- Do you think the course has had an impact on the learning environment at your workplace today? If so, how?
- Has the course influenced your own learning today? If so, how?
