# Supplementary material for: Paediatric on-call consultants’ learning within and beyond the objectives of a coherent CPD program
Source: BMC Med Educ. 2022 Dec 14;22:865. doi: 10.1186/s12909-022-03895-6 (PMC9749295; doi:10.1186/s12909-022-03895-6)
Supplement: Supplementary file 2 — Additional file 2: Supplement Table B. An example of the analysis procedure for the interpretation of the transcribed interview text, using qualitative content analysis. [file 12909_2022_3895_MOESM2_ESM.docx]

Supplement **Table B** An example of the analysis procedure for the interpretation of the transcribed interview text, using qualitative content analysis

| **Meaning unit** | **Condensed meaning unit** | **Condensed meaning units** – interpretation of the underlying meaning | **Codes** | |
| --- | --- | --- | --- | --- |
|  |  |  | **What**  was learned | **How**  it was learned |
| “Going through all the areas again theoretically, reading a lot, repeating it, listening to other people’s ideas and studying new data has given me a sense of confidence. Not the way it was when I was studying in the 1980s and 1990s, because that’s a long time ago. Quite a lot has happened since then”. | Confidence  Studying new, updated knowledge  Repeating and listening to other people’s ideas | Studying new data  Repeating  Confidence  Defining, updating, and extending field of knowledge | Confidence  Updated knowledge | Reading, repeating, and studying other people’s ideas |
